# Impact of anesthesia type on postoperative pain and outcomes in primary hip and knee arthroplasty: a retrospective register analysis

**DOI:** 10.1186/s12871-025-03127-6

**Published:** 2025-05-29

**Authors:** Keno Sponheuer, Diana Becker-Rux, Stephan Scheike, Lukas Barsch, Christina Pempe, Christian Pfrepper, Andreas Roth, Robert Werdehausen

**Affiliations:** 1 Department of Anesthesiology and Intensive Care Medicine, Medical Faculty, University of Leipzig, Liebigstr. 20, Leipzig, 04103 Germany; 2 Present Address: Department of Anesthesiology and Intensive Care Medicine, Charité - Universitätsmedizin Berlin, Corporate Member of Freie Universität Berlin, Humboldt-Universität zu Berlin, and Berlin Institute of Health, Campus Benjamin Franklin, Berlin, Germany; 3 Department of Orthopedics, Trauma and Plastic Surgery, Medical Faculty, University of Leipzig, Leipzig, Germany; 4 Division of Hemostaseology, Department of Hematology, Cellular Therapy and Hemostaseology, Medical Faculty, University of Leipzig, Leipzig, Germany; 5 Department of Anesthesiology and Intensive Care Medicine, Medical Faculty, University of Magdeburg, Leipziger Str. 44, Magdeburg, 39120 Germany

**Keywords:** Endoprosthetic joint replacement, Neuraxial anesthesia, Single-sided spinal anesthesia, General anesthesia, Fast-track surgery, Length of hospital stay, Postoperative pain management

## Abstract

**Background:**

This study explores the effects of single-sided spinal versus general anesthesia on patients undergoing hip and knee arthroplasty within a fast-track surgery environment. Although many studies suggest better outcomes with neuraxial anesthesia in lower extremity joint replacement, its benefits in fast-track surgery remain unclear.

**Methods:**

A retrospective analysis was performed on data derived from 283 patients. We focused on patients who had undergone primary, elective, and unilateral endoprosthetic fast-track hip or knee joint replacement surgeries between May 15, 2019, and November 30, 2020. The primary objective was to ascertain the correlation between the type of anesthesia and several postoperative parameters, including pain intensity, analgesia requirements, incidence of postoperative nausea and vomiting, perioperative process times, and hospital length of stay, based on the collected data.

**Results:**

Our findings indicated no difference in pain intensity and patient satisfaction between the general (n = 195) and single-sided spinal anesthesia (n = 61) groups at the first postoperative day. However, a notable difference was observed in the recovery room, with patients under spinal anesthesia requiring significantly less piritramide than those after general anesthesia. Both groups had a similar incidence of postoperative nausea and length of hospital stay.

**Conclusions:**

When analyzed retrospectively, the type of anesthesia applied is not associated with an increased risk of postoperative pain, postoperative nausea and vomiting, or prolonged hospital stay. Overall, neither anesthesia method outperforms the other concerning patient satisfaction, emphasizing the importance of patient preference and individual risk factors in the decision-making process.

## Introduction

Endoprosthetic joint replacements, employed for hip and knee joint arthrosis, can be conducted under spinal or general anesthesia, each carrying their own benefits and potential drawbacks. Past cohort studies indicate fewer complications, hospital stays, transfusion needs, and reduced mortality with spinal anesthesia for elective hip and knee arthroplasty [1–5]. The ICAROS consensus recommendation from 2019 advocates neuraxial anesthesia for primary elective hip and knee replacements [6].

However, advancements in perioperative medicine and fast-track surgery may have lessened the advantages of spinal anesthesia. Fast-track protocols, incorporating early postoperative mobilization and multimodal pain management, have decreased thromboembolic event rates [7, 8] and various postoperative complications [9], positively affecting postoperative morbidity and hospital stay duration [10]. In fact, a randomized-controlled study discovered no mortality difference at 60 days post-surgery in proximal femur fracture patients based on anesthesia type [11].

Fast-track surgery, introduced in the 1990s, has become a cornerstone of orthopedic arthroplasty. It aids in reducing hospital stays without increasing readmissions, reoperations, or mortality [12, 13], with patients undergoing hip replacements in Denmark having a median hospital stay of just 3 days [14]. This surgical approach employs either short-acting general or spinal anesthesia and incorporates minimally invasive surgical techniques, reducing operative tissue trauma and promoting early mobilization.

Unfortunately, postoperative pain remains prevalent, experienced by around 75% of surgical patients, with 30% reporting moderate-to-severe acute pain [15–19]. Fear of postoperative pain can deter patients from opting for endoprosthetic care [20]. Inadequate pain management can delay mobilization, lengthen hospital stays [21], and potentially contribute to chronic pain syndrome [22].

A retrospective cohort study discovered that neuraxial procedures compared to general anesthesia led to a reduced risk of major complications, need for intensive care, and prolonged hospital stays [23]. Yet, general anesthesia, which is considered safe [24], enables patients to mobilize swiftly after surgery, crucial in a fast-track protocol [25].

Our study aims to retrospectively determine if the type of anesthesia affects postoperative pain intensity, patient satisfaction with pain management on the first postoperative day, and other perioperative outcomes in hip and knee arthroplasty at Leipzig University Hospital (UKL). We also examine postoperative opioid requirements, incidence of postoperative nausea and vomiting, perioperative process times, and length of hospital stay.

## Methods

### Study design and timeline

This research study leverages the data from the Quality Improvement in Postoperative Pain Management (QUIPS) registry. The study was approved by the Ethical committee of the University of Leipzig (reference 241/18-lk), and conducted according to the Declaration of Helsinki. Informed consent was obtained from all subjects and/or their legal guardian(s). The analysis focuses on 283 patients who underwent elective, unilateral, primary endoprosthetic surgery of either the hip or knee joint from May 15, 2019, to November 30, 2020.

### Selection criteria

We included patients who underwent elective, primary, and unilateral hip or knee joint arthroplasty, consented to participate in the QUIPS registry, completed a survey on their first postoperative day, and were aged 18 or above. We excluded patients who had revision surgery due to joint infection, loosening or prosthetic material wear, elective re-implantation of a joint prosthesis, endoprosthetic treatment due to fractures, acetabular or femoral neck fractures needing secondary hip arthroplasty, or repositioning osteotomies on the involved joint. Additionally, cases with pre-existing foreign materials, such as dynamic hip screws, duohead prostheses, or osteosynthesis material, were also excluded.

### Patient group

Preoperative identification of all patients was carried out by the study team from the Department of Anesthesiology and Intensive Care who reviewed the surgical plan. During the study period, a total of 386 patients scheduled for hip or knee surgery were identified. After applying the inclusion and exclusion criteria, 283 patients remained who were scheduled for primary elective joint replacement of the hip and knee. The decision on whether to use general or spinal anesthesia was made in collaboration with the patients (shared decision-making). Cases with insufficient spinal anesthesia or those who received additive peripheral nerve block were excluded from this analysis.

### Data Collection—QUIPS project

The benchmarking project “Quality Improvement in Postoperative Pain Therapy”(QUIPS), registered in the German Register of Clinical Studies (DRKS00006153; https://www.dkrs.de; Date of Registration: 2014–05–12) [26], served as the primary source of data collection. The QUIPS project has been instrumental in enhancing the quality of care across most participating clinics [27].

Data collection involved patients completing a validated questionnaire on their first postoperative day. These data were collected for continuous quality assurance via internal and external benchmarking, with the goal of enhancing postoperative pain treatment. The QUIPS database benchmark server (https://bms.med.uni-jena.de/quips, Takwa GmbH, Erfurt, Germany) was used to extract the datasets. Additional parameters were obtained through the hospital information system (HIS) SAP (SAP Deutschland SE & Co. KG, Walldorf, Germany) and the patient data management system (PDMS) COPRA5 (COPRA System GmbH, Berlin, Germany).

### Statistical analysis

Statistical analysis was completed utilizing IBM SPSS® Statistics for Windows, version 27.0 (IBM Corp., Armonk, New York, USA) and GraphPad Prism, version 9 (GraphPad Software, San Diego, California, USA). GraphPad Prism was also used to generate graphical representations and box-plot diagrams.

In terms of data description, we employed descriptive statistics methods to describe the dataset, including the computation of absolute and relative frequencies, and position parameters such as the mean and median with corresponding standard deviation.

We employed the Pearson chi-square test to study the relationship between two categorical variables. In the event of an expected cell frequency less than 5, we utilized Fisher’s exact test. For comparisons of means, the Mann–Whitney U test was applied for nonparametric variables, while for metric-scaled variables, we used Welch’s T-test, as no advantages were observed in testing the data for normal distribution and variance homogeneity first.

The influence of ASA classification on hospital length of stay was examined using a Welch ANOVA, followed by the Games-Howell post-hoc test for further comparison between the three groups (ASA I-III).

We set the significance level at *p* < 0.05 and categorized the *p* value into three groups: *p* < 0.05; *p* < 0.01; and *p* < 0.001.

### Anesthesia procedures

Patients undergoing elective endoprosthetic surgery on the hip or knee joint had a choice between general and spinal anesthesia. We formed two comparison groups of all patients: those who received general anesthesia and those who received spinal anesthesia.

For general anesthesia, the management protocol recommended the use of short-acting drugs to facilitate rapid postoperative recovery. Overall, 221 procedures were performed under general anesthesia. Balanced anesthesia with sevoflurane or desflurane was used in 204 cases whereas Total Intravenous Anesthesia (TIVA) was used in 16 cases (one patient received a study medication as part of a blinded trial). Sufentanil was used in 153 cases whereas remifentanil was the main opioid in 68 cases.

For spinal anesthesia, we primarily aimed to establish unilateral spinal anesthesia. After administering the local anesthetic (routinely 2.0 to 2.5 mL of hyperbaric bupivacaine 0.5%) slowly at a rate of approximately 1 ml/min, the patient was instructed to remain in lateral position for 15–20 min to ensure adequate fixation time. Hyperbaric bupivacaine was used in 96.1% of patients, with an average dose of 13.0 mg. In cases of partial or complete failure of spinal anesthesia, resulting in failure to achieve an adequately comfortable and pain-free operative condition, routine administration of general anesthesia was performed by hospital protocol for safety reasons. No second attempts at spinal anesthesia were made.

### Fast-track protocol and pain management

Main changes in the fast-track concept were initiation of preoperative patient education and active participation in the recovery process, local infiltration analgesia, minimally invasive surgery, omission of surgical drains, early mobilization on the day of joint replacement and postoperative pain treatment with oral retarded hydromorphone starting in the recovery room. In our study, management of 217 (76.7%) out of a total of 283 patients fully adhered to the protocol as intended. This included the absence of regular premedication with benzodiazepines. The fast-track protocol advised the administration of dexamethasone and ondansetron for PONV prophylaxis in all patients without a specific risk stratification and regardless of the anaesthetic procedure. To reduce blood loss, potential need for blood transfusions and the development of hematoma patients without contraindications received 1 g of tranexamic acid 30 min before incision.

The applied fast-track scheme at our institution included local infiltration analgesia as the primary approach. For local infiltration anesthesia (LIA), ropivacaine 0.2% was administered during surgery. The amount was based on the surgeon’s judgement with a maximum dose of 300 mg (150 mL) as upper limit. This approach has been proven to provide effective and even superior postoperative analgesia compared to regional blocks without time delay, no need for additional pain catheters and no risk of postoperative motor blocks [28].

As part of the multimodal pain therapy concept, patients received a non-opioid analgesic at the end of the procedure. Paracetamol or metamizole were given in 252 patients (89.0%). After surgery, pain therapy was rapidly oralised to circumvent the typical side effects of i.v. opioid administration beginning with the use of oral hydromorphone in the recovery room. Hydromorphone was the standard opioid for patient care also in the following treatment on the surgical wards. Standard criteria by hospital protocol for discharge from the anesthesia recovery unit included stable and adequate vital parameters (breathing, oxygenation, heart rate, blood pressure, core temperature, consciousness), intact protective reflexes, acute pain NRS < 3, absence of PONV and active bleeding. To promote patient independence, oral fluids were offered in the recovery room and patients were motivated to start moving the operated extremity without the use of movement bans.

After discharge from the anesthesia recovery unit, early mobilization was pursued by physiotherapists and nursing staff as trained with patients preoperatively on the day of surgery. To facilitate mobilization surgical drainage and urinary catheterization was omitted routinely.

Further pain treatment was conducted at the orthopedic wards and not controlled by the anesthesia department. In 93.3% of patients, further treatment included oral opioids, primarily retarded oral hydromorphone (84.8%). Unretarded oral hydromorphone was applied in 50.2% of patients for acute pain treatment.

Our objective was to improve postoperative pain management, and this was pursued by adopting the fast-track protocol and considering the specific protocols outlined in our study design.

## Results

### Demographic data and patient characteristics

**Fig. 1:**
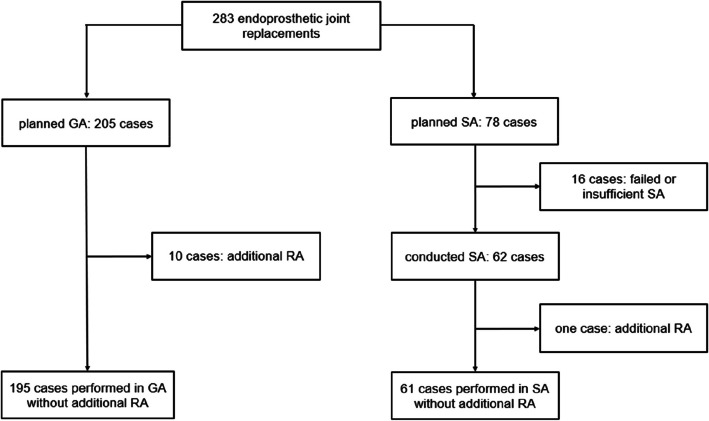
Flow chart: representation of planned and actually performed anesthesia procedures. GA, general anesthesia, SA, spinal anesthesia. RA, regional anesthesia

Among the 283 patients examined, general anesthesia was scheduled in 205 patients (72.4%) and spinal anesthesia in 78 patients (27.6%) (Fig. 1). General anesthesia was supplemented with peripheral pain catheters in 10 cases, which were therefore not included into our analysis. A change of procedure to general anesthesia was necessary due to insufficient spinal anesthesia in 16 cases.

The majority of patients fell under the American Society of Anesthesiologists (ASA) II classification, constituting 70.3% of cases. The distribution of ASA III patients was not significantly different across anesthesia types (21.7% general anesthesia, 30.7% spinal anesthesia; Pearson chi-square test; *p* = 0.144).

Surgery duration averaged 64 min for hip arthroplasty and 82 min for knee arthroplasty. The distribution of anesthesia types was not significantly different across hip and knee arthroplasties (77.7 vs. 79.8% general anesthesia, 22.3 vs. 20.9% spinal anesthesia, respectively; Pearson chi-square test; *p* = 0.793). Notably, 76.7% (217 patients) were managed with full adherence to the fast-track protocol (Table 1).

**Table 1:** Demographic data and patient characteristics (total collective). Data are given as absolute numbers (and relative proportions, %) or means with standard deviation. The definition of weight classification is given in the Methods section

	**Total**	**Hip**	**Knee**	**GA**	**SA**
	*n* = 283	*n* = 197	*n* = 86	*n* = 221	*n* = 62
**Age** (years)	67 ± 11	67 ± 11	67 ± 10	66 ± 11	71 ± 10
**Male/Female **(n, %)	142 (50.2%)/141 (49.8%)	97 (49.2%)/100 (50.8%)	45 (52.3%)/41 (47.7%)	106 (48.0%)/115 (52.0%)	36 (58.1%)/26 (42.0%)
**General/Spinal anesthesia** (n, %)	221 (78.1%)/62 (21.9%)	153 (77.7%)/44 (22.3%)	68 (79.1%)/18 (20.9%)		
**ASA status** (n, %)					
I	17 (6.0%)	16 (8.1%)	1 (1.2%)	15 (6.8%)	2 (3.2%)
II	199 (70.3%)	136 (69.0%)	63 (73.3%)	158 (71.5%)	41 (66.1%)
III	67 (23.7%)	45 (22.9%)	22 (25.5%)	48 (21.7%)	19 (30.7%)
**OP duration** (minutes)	70 ± 22	64 ± 20	82 ± 19	70 ± 22	68 ± 20
**Full adherence to fast-track protocol** (n, %)	217 (76.7%)	155 (78.7%)	62 (72.1%)	165 (74.7%)	52 (83.9%)

*Hip* total hip arthroplasty, *knee* knee arthroplasty, *GA* general anesthesia, *SA* spinal anesthesia

### Local infiltration anesthesia

Additional local infiltration anesthesia (LIA) was performed as intended by the fast-track protocol in 81.5% of patients in the general anesthesia group and in 83.6% of patients in the spinal anesthesia group (Table 2).

**Table 2:** Use of local infiltration anesthesia (LIA)

	**LIA + **	**LIA -**	**Total**
**Type of Anesthesia (procedures**			
**General anesthesia**	159 (81.5%)	36 (18.5%)	195
**Spinal anesthesia**	51 (83.6%)	10 (16.4%)	61
**Operation type**			
**Hip arthroplasty**	157 (84.4%)	29 (15.6%)	186
**Knee arthroplasty**	53 (75.7%)	17 (24.3%)	70

Proportions (n, %) of the final collective of patients from the general and spinal anesthesia groups (*n* = 256) according to whether local infiltration anesthesia was used intraoperatively

Top: Subdivision by anesthesia type. Bottom: Subdivision by operation type

### Postoperative pain

On the first postoperative day, patients were asked about their pain and provided information on minimum, maximum, and exertion intensity.

**Fig. 2:**
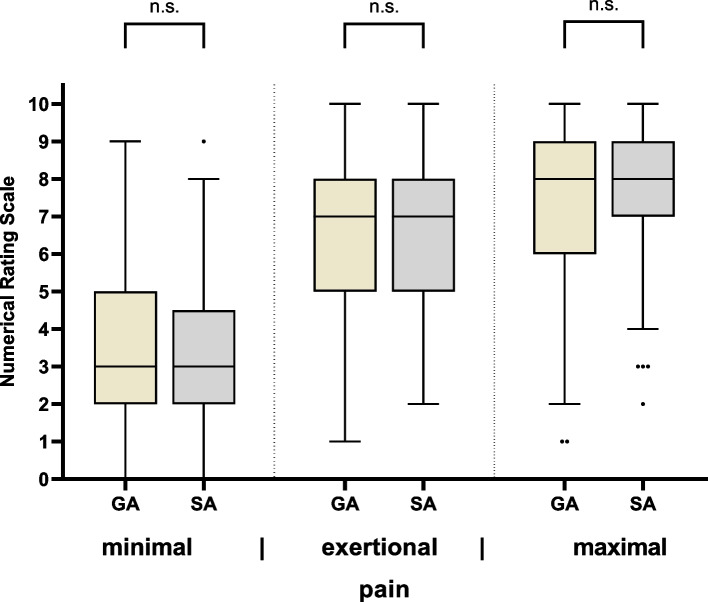
Intensity of pain on the first postoperative day depending on the anesthesia procedure. Displayed as a box-plot diagram (median, standard deviation and outliers as dots) with subdivision according to anesthesia procedure Representation of minimal, exertional, and maximal pain on the Numerical Rating Scale. n.s.—not significant. GA, general anesthesia (*n* = 195). SA, spinal anesthesia (*n* = 61)

NRS values were compared depending on type of surgery and the anesthetic procedure. Median NRS scores were comparable between hip and knee replacement surgeries, with values of 3 vs. 3.5 at rest (minimum), 7 vs. 7 during exertion, and 8 vs. 8 at peak intensity (maximum). Because of the similarity in the primary outcome pain scores and to improve clarity and statistical power, data from both surgery types were pooled for all subsequent analyses. There was no significant difference between the group that had received general anesthesia alone (*n* = 195) and the group that had received spinal anesthesia alone (*n* = 61) for all three pain categories (Mann–Whitney U test; minimum intensity: *p* = 0.508; intensity on exertion: *p* = 0.097; maximum intensity: *p* = 0.547; Fig. 2).

The median value of patient satisfaction with pain therapy was 7 (on a scale from 0 to 10) in both groups and did not differ significantly (*p* = 0.394).

The amount of hydromorphone administered on the surgical wards between discharge from the recovery room and the interview on postoperative day 1 did not differ significantly. Patients in the general anesthesia group were given 4.9 mg and patients in the spinal anesthesia group 4.8 mg of hydromorphone (*p* = 0.986).

### Postoperative opioid use in the recovery room

**Fig. 3:**
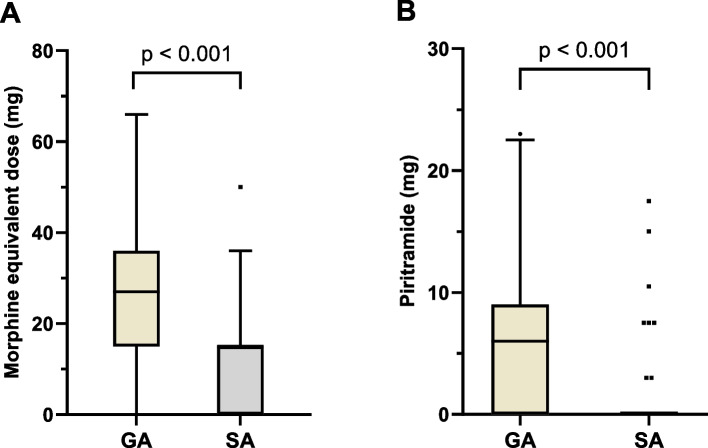
Administered dose of opioid analgesics in the recovery room. Morphine equivalent dose (**A**) and amount of piritramide (**B**). Data given as box-plot diagrams (median, standard deviation and outliers as dots) with subdivision according to anesthesia procedure. GA, general anesthesia (*n* = 195). SA, spinal anesthesia (*n* = 61)

Patients in the general anesthesia group received a mean of 25.5 mg of oral morphine equivalents in the recovery room, whereas a mean of 12.9 mg was administered in the spinal anesthesia group. There was a statistically significant difference in the dose of oral morphine equivalents administered, with an average of 12.6 mg lower in the spinal anesthesia group (Welch t-test, 95% confidence interval [9.1, 16.1]; *p* < 0.001; Fig. 3A).

No opioids were administered in the recovery room in 21 of the 195 patients (10.8%) in the general anesthesia group and in 19 of the 61 patients (31.1%) in the spinal anesthesia group.

In the general anesthesia group, 143 of the 195 patients (73.3%) and in the spinal anesthesia group, 8 of 61 patients (13.1%) received piritramide intravenously as a rescue medication. This difference was statistically significant (Pearson chi-square test, χ^2^ (1) = 69.65; *p* < 0.001, φ = −0.522 corresponding to a high effect size). The mean dose of piritramide administered was 6.6 mg for general anesthesia and 1.2 mg for spinal anesthesia. The comparison of the total amount of intravenously administered piritramide showed a statistically significant difference. (Welch t-test, 95% confidence interval [4.2, 6.6]; *p* < 0.001) (Fig. 3B).

**Fig. 4:**
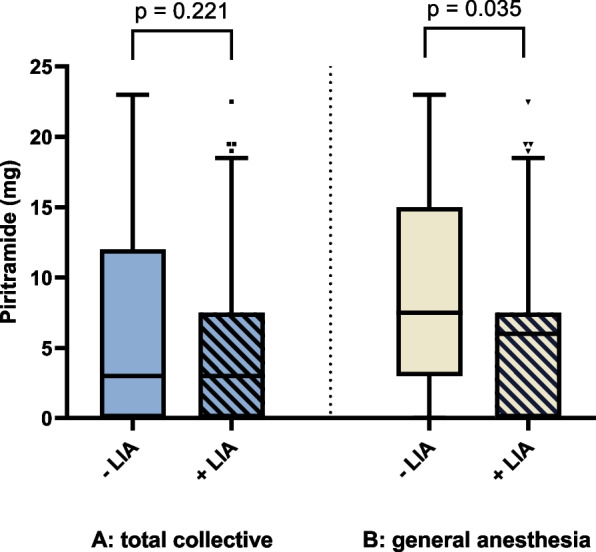
Administered intravenous amount of piritramide in the recovery room depending on whether local infiltration anesthesia was performed. **A** For the total collective. **B** For the general anesthesia group. Box-plot diagram with data given as median, standard deviation and outliers as dots. Overall collective (n = 283). General anesthesia group (*n* = 195). – LIA, no local infiltration anesthesia performed. + LIA, local infiltration anesthesia performed

In the total patient collective (*n* = 283; no consideration of anesthesia procedure), the use of local infiltration anesthesia (LIA) did not result in a statistically significant reduction in piritramide dose in the recovery room (*p* = 0.221). The mean dose of piritramide in the group without LIA (57 patients) was 6.1 mg and in the group with LIA (226 patients) 4.8 mg. In the general anesthesia group (*n* = 195), a statistically significant difference was observed when patients additionally received LIA. Here, the average piritramide dose was 2.7mg lower compared to the patients who had general anesthesia alone (Welch t-test, 95% confidence interval [0.2, 5.2], *p* = 0.035) (Fig. 4).

There were no statistically significant differences detected in NRS scores for minimum, maximum, and exertion pain on the first postoperative day, when comparing patients with or without local infiltration anesthesia (Mann–Whitney U test, *n* = 256, group 1: no LIA received, group 2: LIA received). The p-values were 0.056 for minimal pain, 0.874 for exertional pain, and 0.925 for maximal pain.

### Frequency of postoperative nausea and vomiting

In the general anesthesia group, PONV prophylaxis was given in 190 of the 195 patients (97.4%). Dexamethasone alone was given in 14 cases, ondansetron alone in 60 cases, and combined dexamethasone and ondansetron in 116 cases.

In the spinal anesthesia group, 43 of the 61 patients (70.5%) received PONV prophylaxis. PONV prophylaxis was given with dexamethasone in 2 cases, with ondansetron in 7 cases, and with both agents combined in 34 cases. There was a statistically significant association between the anesthetic procedure and the frequency of PONV prophylaxis administered (Pearson chi-square test, *p* < 0.001).

The proportion of patients with PONV in the recovery room who required drug therapy was not significantly different between the two anesthetic regimens (Pearson Chi-squared test, *p* = 0.284). On the first postoperative day, 47 of the 195 patients (24.1%) in the general anesthesia group and 14 of the 61 patients (23.0%) in the spinal anesthesia group reported having suffered from nausea. There was no statistically significant difference in the incidence of postoperative nausea on the first postoperative day depending on the anesthetic procedure (Pearson chi-square test, *p* = 0.854).

The rate of postoperative nausea was 15% in the cohort of patients who received spinal anesthesia with 2-drug PONV prophylaxis (*n* = 34) and 39% in the cohort of patients who received spinal anesthesia without drug PONV prophylaxis (*n* = 18). The difference was not significant by Fisher’s exact test (*p* = 0.082) (Table 3).

**Table 3:** Incidence of postoperative nausea and vomiting (PONV) in relation to the anesthetic procedure and in relation to the type of drug PONV prophylaxis

**General Anesthesia**	**PONV**	**no PONV**	**Total**
no PONV prophylaxis	1 (20%)	4 (80%)	5
Dexamethasone	3 (21%)	11 (79%)	14
Ondansetron	10 (17%)	50 (83%)	60
Dexamethasone and ondansetron	33 (28%)	83 (72%)	116
Total	47 (24%)	148 (76%)	195
**Spinal Anesthesia**	**PONV**	**no PONV**	**Total**
no PONV prophylaxis	7 (39%)	11 (61%)	18
Dexamethasone	0 (0%)	2 (100%)	2
Ondansetron	2 (29%)	5 (71%)	7
Dexamethasone and ondansetron	5 (15%)	29 (85%)	34
Total	14 (23%)	47 (77%)	61

### Length of hospital stay

The choice of anesthesia procedure had no statistically significant effect on the length of hospital stay. The mean length of stay was 179.5 h (7.5 days) in the general anesthesia group and 185.5 h (7.7 days; Welch t-test, *p* = 0.784) in the spinal anesthesia group.

The type of joint replacement (hip joint endoprosthesis or knee joint endoprosthesis) had no statistically significant effect on the average length of hospital stay (Welch t-test, *p* = 0.969): This was 179.4 h for patients operated on the hip joint and 178.8 h for patients operated on the knee joint.

### ASA classification

**Fig. 5:**
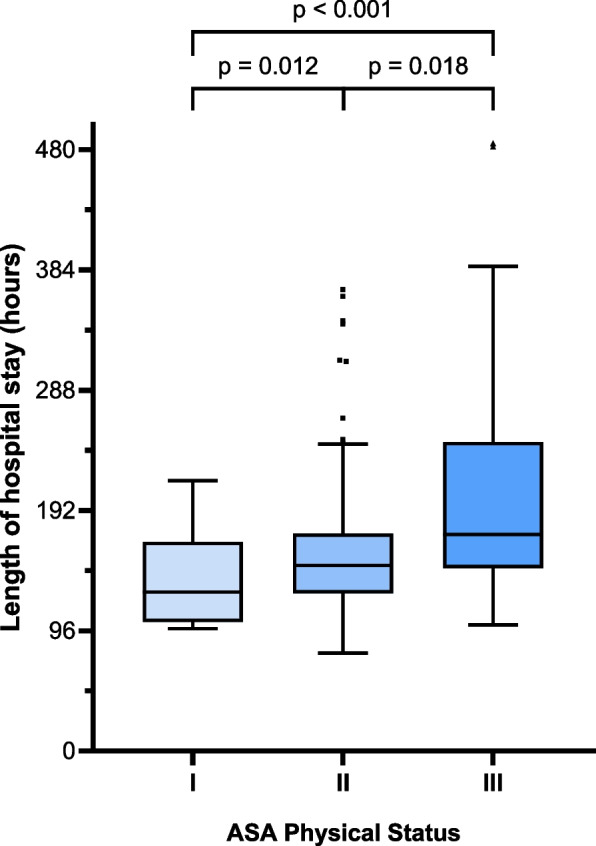
Length of hospital stay as a function of comorbidity. Data are given Median, standard deviation and outliers as dots. Scale limited for better overview, the outliers of ASA classes II and III are not depicted completely. ASA status: classification of the *American Society of Anesthesiologists.* Analysis of the total collective (*n* = 283; ASA I: *n* = 17; ASA II: *n* = 199; ASA III: *n* = 67)

The influence of ASA classification on hospital length of stay was investigated. There was a significant difference of the mean values in the total collective (Welch-ANOVA, *p* < 0.001), as well as between the three groups (ASA I, II and III) among each other in the post-hoc test according to Games-Howell (Fig. 5).

### Perioperative process times and postoperative monitoring

The anesthesia induction time (time between the onset of anesthesia and the time of release) was statistically significantly lower in the general anesthesia group (13.7 min) than in the successfully performed spinal anesthesia group (24.2 min), (Welch t-test, *p* < 0.001, 95% confidence interval [7.1, 13.9]).

The time for anesthesia lead-out was 3.6 min in the spinal anesthesia group and 7.7 min in the general anesthesia group. The mean difference was 4.1 min and was statistically significant (Welch t-test, *p* < 0.001, 95% confidence interval [−6.2, −1.9]).

Anesthesia-controlled time refers to the anesthesiologist’s involvement in patient care and includes the total time for anesthesia induction and emergence. The anesthesia-controlled time was 40.8 min in the general anesthesia group and 49.7 min in the spinal anesthesia group (Welch t-test, *p* < 0.001; 95% confidence interval [3.9, 14.4]).

### Postoperative length of stay in the recovery room

**Fig. 6:**
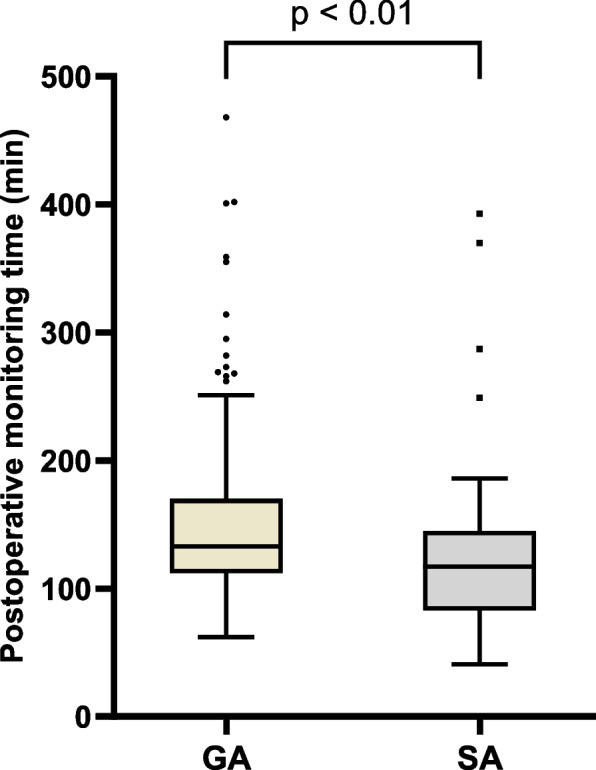
Postoperative monitoring time in the recovery room. Median, standard deviation and outliers as dots are given according to anesthesia procedure. Scale capped at 500 min, thus one outlier in the general anesthesia group is not depicted. GA, general anesthesia (*n* = 195). SA, spinal anesthesia (*n* = 61)

The spinal anesthesia group showed an average of 28.1 min less time spent in the recovery room (Welch t-test, mean general anesthesia group: 153.8 min; mean spinal anesthesia group 125.7 min; *p* < 0.01), when the same standard discharge criteria were applied to both groups. The median time in the general anesthesia group was 133 min and 117 min in the spinal anesthesia group. A right-skewed distribution is present for both anesthetic procedures, which is more pronounced in the general anesthesia group and explains the greater deviation of the mean from the median in the general anesthesia group (skewness general anesthesia group 6.484; skewness spinal anesthesia group: 2.272) (Fig. 6).

## Discussion

Our retrospective analysis explored the ideal anesthetic technique for primary lower extremity arthroplasty in adults. The anesthetic technique had no significant impact on pain intensity on the first postoperative day. Patient satisfaction with the pain treatment was comparable. Patients given spinal anesthesia received less intravenous piritramide in the recovery room, presumably due to the analgesic effect of intrathecal drugs. Those findings are in line with the current literature [29]. On the one hand, we did not see a positive prolonged effect on postoperative pain after the initial benefit of spinal anesthesia. On the other hand, we did not see higher pain scores due to previously described undertreatment of initially pain free patients in the spinal anesthesia group after spinal block resolution [30].

Local infiltration anesthesia (LIA) with general anesthesia reduced piritramide use in the recovery room but did not affect postoperative pain on day one. The average length of hospital stay was approximately 180 h (~ 7.5 days), with slight variations across ASA status groups.

The results from the patient survey were obtained via the validated QUIPS project questionnaire. The implementation of fast-track surgery at UKL around the time of data collection in 2019 may have caused some inconsistencies. Furthermore, the study was retrospective without randomization, leading to an uneven distribution between general and spinal anesthesia groups. The sample size was also limited due to protocol changes in December 2020.

Our findings align with other studies suggesting that patients receiving spinal anesthesia require less opioids in the recovery room [25, 31–33]. Data collection in the QUIPS project does not include structured analysis of pain ratings in the recovery room. However, the use of piritramide was triggered only by pain on the numerical rating scale exceeding 3, therefore the amount of piritramide administration serves as a satisfactory surrogate parameter for postoperative pain.

Current recommendations favor the use of LIA for knee arthroplasty, but not hip arthroplasty [34]. This is due to insufficient evidence for LIA in hip arthroplasty [35]. LIA may primarily help in reducing opioid analgesics in the first 12 h with general anesthesia [36].

Another evidence based approach to improve pain control and reduce opioid requirements is the administration of higher doses (e.g. 8–10 mg) of dexamethasone which was not yet part of the standard protocol during our study [29]. Optimal prophylaxis of postoperative nausea and vomiting (PONV) has previously been shown to be effective to reduce the incidence of PONV after spinal anaesthesia with morphine [37]. Regional anesthesia is usually favored over general anesthesia to minimize PONV risk [38]. Our study found no difference in PONV incidence between anesthesia procedures, with 20 to 25% of patients in both groups being affected. Our findings therefore suggest adequate PONV prophylaxis for every patient undergoing spinal anesthesia.

The average anesthesia induction time was 11.5 min shorter for the general anesthesia group. This, together with a considerably high rate of failed or insufficient cases of spinal anesthesia, may be caused by the fact that most cases were performed by early-stage trainees. However, a recently published prospective observational study revealed similar “real world data” with a failure rate of 22.4% [39]. Several factors were associated with failed spinal anesthesia that were also present in our population like work experience of the anesthetist < 2 years, hyperbaric local anesthetic drug, a reduced dose of bupivacaine of ≤ 10 mg (25% of injections in our study) and the renouncement of adjuvants. Moreover, puncture attempts in the lateral decubitus position are associated with higher failure rates [40]. It is imperative to consider the potentially higher failure rate associated with unilateral spinal anesthesia including a slow injection technique, which must be weighed against the benefits, which include hemodynamic stability and facilitated postoperative mobility. Furthermore, spinal anesthesia in this patient population was potentially more challenging due to calcified ligaments (average age 71 years) and obesity, complicating palpation, landmark identification and increasing the depth of puncture.

Patients in the spinal anesthesia group had significantly shorter recovery room stays.

The hospital length of stay in Germany for primary hip and knee joint implantation has decreased significantly since 2009. Our study found no difference in length of hospital stay based on the anesthesia procedure. While a detailed analysis of the possible reasons for the overall comparatively long hospital stay despite management with a fast-track protocol is beyond the scope of our study, one may argue that most of the included patients could have been discharged earlier, if post-hospital care would be available as in other centers with shorter hospital stays (e.g., the United States). Moreover, ASA classification is a rough division and many of our ASA III patients do have more than one severe comorbidity (e.g., intermittent continuous renal replacement therapy, stable but severe pulmonary disease) without fulfilling the criteria for ASA IV. Those patients are generally not operated on in smaller, specialized, high-volume centers.

As an observational study, this research did not intervene in medical procedures or decision-making. Consequently, there is potential for confounding and selection bias, as patients were not randomized but instead chose their anesthetic procedure through a patient-centered decision-making process. Moreover, also comorbidities might have changed the choice of anesthetic technique. The case numbers between the general and spinal anesthesia groups were significantly different, which might affect the results. Favorably, patients could be included into our analysis, that would have been excluded in case of a randomized study (e.g. those with contraindications or firm preference for one type of anesthesia). Therefore, one may argue that our data set may be more representative.

Furthermore, this study lacked an extended postoperative follow-up. Complications, serious adverse events, data on postoperative mobilization, delirium and mortality were not part of data collection.

Pain management on the surgical wards after the recovery room period was provided by the surgical ward physicians, but without the use of a standardized analgesia study protocol. Both anesthesia groups received equal amounts of hydromorphone postoperatively. Pain being a subjective perception influenced by numerous factors adds to the complexity. The inconsistent adherence to the fast-track protocol with regard to local infiltration anesthesia (LIA) also represents a limitation.

Overall, neither anesthesia method outperformed the other concerning postoperative pain and patient satisfaction, emphasizing the importance of patient preference and individual risk factors in the decision-making process.

## Data Availability

The data that support the findings of this study are available on request from the corresponding author, R.W. The data are not publicly available due to their containing information that could compromise the privacy of research participants.
